# Counting the Ways That Aboriginal and Torres Strait Islander Older People Participate in Their Communities and Culture

**DOI:** 10.1093/geronb/gbae100

**Published:** 2024-05-31

**Authors:** Joanne Nicole Luke, Dawn Bessarab, Kate Smith, Dina LoGiudice, Leon Flicker, Lianne Gilchrist, Briony Dow, Jeromey Temple

**Affiliations:** Centre for Health Policy, School of Population and Global Health, Faculty of Medicine, Dentistry and Health Sciences,The University of Melbourne, Melbourne, Victoria, Australia; Centre for Aboriginal Medical and Dental Health, Medical School, The University of Western Australia, Perth, Western Australia, Australia; Centre for Aboriginal Medical and Dental Health, Medical School, The University of Western Australia, Perth, Western Australia, Australia; Department of Medicine, Faculty of Medicine, Dentistry and Health Sciences, The University of Melbourne, Melbourne, Victoria, Australia; Western Australian Centre for Health and Ageing, Medical School, University of Western Australia, Perth, Western Australia, Australia; Centre for Aboriginal Medical and Dental Health, Medical School, The University of Western Australia, Perth, Western Australia, Australia; Department of Medicine, Faculty of Medicine, Dentistry and Health Sciences, The University of Melbourne, Melbourne, Victoria, Australia; National Ageing Research Institute, Melbourne, Victoria, Australia; Centre for Health Policy, School of Population and Global Health, Faculty of Medicine, Dentistry and Health Sciences,The University of Melbourne, Melbourne, Victoria, Australia; (Social Sciences Section)

**Keywords:** Cultural capital, Cultural determinants, Indigenous, Social capital

## Abstract

**Objectives:**

This study aimed to determine the proportion of older Aboriginal and Torres Strait Islander peoples participating in cultural events and activities and determine the demographic and sociocultural characteristics associated with participation.

**Methods:**

The Australian Bureau of Statistics National Aboriginal and Torres Strait Islander Social Survey (2014–2015) was used to measure the prevalence of participation in cultural events and activities. Multivariate logistic regression models were used to measure associations. Sociocultural factors were selected by matching survey items to the 12 sociocultural factors described in the Good Spirit Good Life Framework, a culturally validated quality-of-life tool for older people.

**Results:**

The majority (62.0%) of survey respondents 45 years and older participated in cultural events (e.g., ceremonies, funerals/sorry business, NAIDOC week activities, sports carnivals, festivals/carnivals) or were involved in organizations. Many (58.5%) also participated in activities (e.g., fishing, hunting, gathering wild plants/berries, arts/crafts, music/dance/theater, writing/telling of stories). In regression models including demographic and cultural variables, participation in cultural events was highest among people living remotely (odds ratio [OR] = 2.71), reporting recognition of homelands (OR = 2.39), identifying with a cultural group (OR = 3.56), and those reporting having a say in their communities (OR = 1.57), with similar odds seen for participation in activities. Participation was inversely proportional to increasing age, with a greater proportion of females participating in events and males in activities.

**Discussion:**

The social lives of older Aboriginal and Torres Strait Islander people were characterized by widespread participation in cultural events and activities. These findings provide important insights into services as they support older people to live a good life.

Aboriginal and Torres Strait Islander people are the First People of territories now recognized as Australia. They represent a diverse population from over 250+ nation groups and geographies, yet share many sociocultural commonalities including shared experience of ongoing colonization. For older Aboriginal and Torres Strait Islander people, much has and continues to change in their lifetime. They are the babies that were birthed on verandas of hospitals, the children raised in country, on missions and reserves, and in the pebbled laneways of suburbs and cities. They include the members of the stolen generation, who are the children forcibly taken from their families by Australian governments, and for many, they are the last generations born under the discriminatory Aboriginal Acts. They are the generation that paved the way as campaigners for equal rights, human rights, Aboriginal self-determination, and land rights, and who have come to live with greater rights. They are now the generation lovingly known to many of us as nan, pop, Aunty, and Uncle, and hold important roles in their families and communities. In this study, we explore this role as we measure how older people participate in their communities and culture.

The contemporary social sciences are dominated by Western-centric theories and frameworks for aging, where concepts of “positive,” “active,” and “successful” aging (Ranzijn, 2010) are emphasized. Although there are benefits to these strengths-based frameworks in that they produce powerful counter-narratives to past research which focused on the burdens and societal economic costs of aging, the cultural location of these frameworks is limited in understanding aging in Indigenous contexts (Ranzijn, 2010). Located in the dominant cultural epistemologies, ontologies, and axiologies, these frameworks assume a universality of Western social norms and experiences of aging (Quayle & Sonn, 2019; Quigley et al., 2022). In Indigenous settings, globally “ageing well” as a “holistic concept enabled by spiritual, physical, and mental wellbeing and on connections to person, place, and culture is central” (Quigley et al., 2022, p. 1) has emerged as a more socially inclusive framework to aging and for measuring aging for this population (Quigley et al., 2022; Ranzijn, 2010).

In Australia, past dominant social demography has tended to present older people’s participation through a Western cultural lens where constructs such as “volunteering” or “caring” are said to provide social capital to older individuals as well as economic benefits to society more broadly (Warburton & Chambers, 2007; Warburton & McLaughlin, 2006). However, in Aboriginal and Torres Strait Islander contexts, such constructs do not accurately conceptualize or measure social participation (Quayle & Sonn, 2019). First, older Aboriginal and Torres Strait Islander people participate differently in broader Australian society, as participation is constrained by processes of colonization which have limited capital and resources to “positively,” “successfully,” or “actively” age (Quigley et al., 2022; Temple et al., 2019). Second, Aboriginal and Torres Strait Islander people participate in their own social communities where participation is not tied to the capitalist economy, rather social capital is acquired at the family and community level where reciprocal benefits come when Aboriginal and Torres Strait Islander older people partake in cultural activities such as fishing, hunting, art and ceremony, promote culture, support family, and provide governance within their communities (Busija et al., 2018; Warburton & Chambers, 2007; Warburton & McLaughlin, 2006). Brough et al. (2006) describe this as “bonding capital,” where Aboriginal and Torres Strait Islander people’s participation within their families and community networks shapes individual and collective well-being that contributes to aging well (Brough et al., 2006).

In recent decades, many qualitative studies (Busija et al., 2018; Coombes et al., 2018; Eades et al., 2021; Gair et al., 2019; Gibson et al., 2020; Smith et al., 2021; Warburton & Chambers, 2007; Warburton & McLaughlin, 2006; Waugh & Mackenzie, 2011) have provided evidence that draws on the rich narrative accounts and voices of Aboriginal and Torres Strait Islander Elders (see Author Note 1) and older people that speak to their sociocultural experiences and realities. These studies describe older peoples’ participation in their culture and communities and describe the important roles and social networks that older people hold and play in the transmission of cultural knowledge as cultural holders and keepers of traditional culture and language, as well as leaders, carers of families, and mentors of youth (Busija et al., 2018; Coombes et al., 2018; Eades et al., 2021; Gair et al., 2019; Gibson et al., 2020; Smith et al., 2021; Warburton & Chambers, 2007; Warburton & McLaughlin, 2006; Waugh & Mackenzie, 2011). Participation is presented in the form of bonding capital where Aboriginal and Torres Strait Islander cultural, family, and community life is presented as integral to establishing strong identities, cultural roles, and relational supports (Busija et al., 2018; Coombes et al., 2018; Eades et al., 2021; Gair et al., 2019; Gibson et al., 2020; Smith et al., 2021; Warburton & Chambers, 2007; Warburton & McLaughlin, 2006; Waugh & Mackenzie, 2011). In recent decades, many qualitative studies (Busija et al., 2018; Coombes et al., 2018; Eades et al., 2021; Gair et al., 2019; Gibson et al., 2020; Smith et al., 2021; Warburton & Chambers, 2007; Warburton & McLaughlin, 2006; Waugh & Mackenzie, 2011) have provided evidence that draws on the rich narrative accounts and voices of Aboriginal and Torres Strait Islander Elders and older people that speak to their sociocultural experiences and realities. These studies describe older peoples’ participation in their culture and communities and describe the important roles and social networks that older people hold and play in the transmission of cultural knowledge as cultural holders and keepers of traditional culture and language, as well as leaders, carers of families, and mentors of youth (Busija et al., 2018; Coombes et al., 2018; Eades et al., 2021; Gair et al., 2019; Gibson et al., 2020; Smith et al., 2021; Warburton & Chambers, 2007; Warburton & McLaughlin, 2006; Waugh & Mackenzie, 2011).

Many of the qualitative studies also provide valuable first-hand insights into social–cultural factors that are unique to older Aboriginal and Torres Strait Islander peoples (Busija et al., 2018; Coombes et al., 2018; Eades et al., 2021; Gair et al., 2019; Gibson et al., 2020; Smith et al., 2021; Warburton & Chambers, 2007; Warburton & McLaughlin, 2006; Waugh & Mackenzie, 2011). These unique factors are also captured in the Good Spirit Good Life Framework, a culturally validated quality-of-life tool developed by Smith et al. (2021) using participatory action research methods (Supplementary Figure 1; Gilchrist et al., 2023; Smith et al., 2021). The Good Spirit Good Life Framework is a strength-based framework for “ageing well” that links and highlights the following 12 sociocultural factors of basic needs, family and friends, country, community, culture, health, respect, elder role, supports and services, safety and security, spirituality, and future planning as important to having a good life (Smith et al., 2021). Having a “Good Life” is essential in promoting healing, strengthening, and protecting the spirit of older people in staying well (Smith et al., 2021).

To date, little quantitative research relating to Aboriginal and Torres Strait Islander people has measured participation in the unique sociocultural contexts for aging. Instead, quantitative research has tended to report on the individual and societal burdens of aging, with a focus on the characteristics and prevalence of individual physical and biological dimensions such as dementia and cognitive impairment (Derrig et al., 2020; Hocking et al., 2019; Lavrencic et al., 2019; Radford et al., 2017; Russell et al., 2021; Smith et al., 2008), disability and frailty (Gubhaju et al., 2015; Hyde et al., 2016, 2019; Lo Giudice et al., 2012; Lukaszyk et al., 2018; Wong et al., 2013), incontinence (Lo Giudice et al., 2012; Smith et al., 2019), chronic disease (McKercher et al., 2014; McNamara et al., 2014; Toelle et al., 2013), psychological distress (Almeida et al., 2014; Mate et al., 2020; Rowland et al., 2021; Shen et al., 2018; Temple, Kelaher, et al., 2020), smoking (Thurber et al., 2021), and the broader demographics of population aging (Lo Giudice et al., 2012; Temple, Wilson, et al., 2020). Although there is increasing research that reports on narrowly defined aspects of Aboriginal sociocultural livelihoods including studies that describe racism (Temple et al., 2019; Temple, Kelaher, et al., 2020), access to aged care and health services (Larke et al., 2020), medications and vaccine equity (Dyda et al., 2019; Page et al., 2019), the role of Aboriginal people as grandparents (Gibberd et al., 2020), childhood adversity and stress (Radford et al., 2017), and self-reported good health (Lavrencic et al., 2020), there remains a gap in quantitative data contributing to a comprehensive picture of the sociocultural lives of older Aboriginal and Torres Strait Islander older people. Such national health and social data are needed to provide an alternative narrative that accurately reflects the cultural values, priorities, and worldviews of Aboriginal Elders and older people. These data and narratives are fundamental to the planning, implementation, and delivery of culturally appropriate health and aged care policy, programs, and services.

Although there are numerous qualitative studies that describe older Aboriginal and Torres Strait Islander people’s participation in their communities and the social contexts important to aging well, there remains a paucity of data quantifying this. In this project, we use data from the Australian Bureau of Statistics (ABS) National Aboriginal and Torres Strait Islander Social Survey, 2014/2015 (NATSISS), one of the few surveys to collect social items that are specific to Aboriginal and Torres Strait Islander people, to answer the research questions:

- How many older Aboriginal and Torres Strait Islander people report participating in cultural events and activities?
- What demographic and sociocultural contexts (as informed by the Good Spirit Good Life Framework) are associated with participation in cultural events and activities? (Smith et al., 2021).

## Method

### Ethics and Research Considerations for Research Involving Aboriginal and Torres Strait Islander Peoples

The University of Melbourne Ethics Committee approved data analysis in August 2021 (Reference: 2021-21631-20948-3). Survey data used in this study were collected under the Census and Statistics Act. Permissions to access data were provided on October 15, 2021, by the ABS under the ABS and Universities Australia agreement. Research proposals were submitted using the ABS DataLab Project Proposal form and approved by the ABS DataLab office and their Aboriginal and Torres Strait Islander group. Approved researchers (J.N. Luke and J. Temple) were provided access to deidentified data accessible through ABS DataLab and were able to share aggregate data with the research team once vetoed by ABS staff to ensure guarantee of data confidentiality.

All research works were conducted in accordance with the National Health and Medical Research Council’s “Australian guidelines for ethical research involving Aboriginal individuals and communities” (National Health and Medical Research Council, 2018). Research priorities were identified by the wider research team, which included Aboriginal and Torres Strait Islander researchers, as well as from the empirical evidence.

### Survey Data

Data were drawn from the NATSISS, a 6-yearly self-reported survey, last conducted by the ABS, an Australian government agency, between September 2014 and June 2015. Survey methodology for the NATSISS are well described, but for brevity, the NATSISS is an Australia-wide survey conducted with Aboriginal and/or Torres Strait Islander people residing in private dwellings in remote and nonremote areas across all Australian States and Territories (Australian Bureau of Statistics, 2019). All respondents provided free and informed consent. Individuals residing in nonprivate dwellings, which include hospitals, hostels, and nursing homes, are excluded from participating in the survey. Of households approached, 73.4% fully or adequately responded.

The NATSISS used a computer-assisted interviewing questionnaire format, where ABS interviewers trained in cultural awareness delivered the questionnaire. In discrete communities and outstations, interviewers were assisted by Aboriginal or Torres Strait Islander advisors who facilitated the interviews. The NATSISS survey questionnaire was developed in consultation with Aboriginal and Torres Strait Islander peak bodies, government departments, and the ABS Advisory Group for Aboriginal and Torres Strait Islander Statistics.

The NATSISS survey instrument included a broad range of demographic and sociocultural measures, encompassing population context, language and culture, social networks, employment, health, and access to services. All variables were self-reported.

### Co-analysis of Data

Participation of Aboriginal and Torres Strait Islander people was centered in this research through a triangulation method with three groups contributing to co-analysis:

1. **The Good Spirit Good Life Elders Governance Group (GSGL Elders Governance Group)** is represented by Aboriginal Elders Aunty Vonita Walley, Aunty Doreen Nelson, and Aunty Doris Getta.
2. **The Good Spirit Good Life qualitative team** included Aboriginal (D. Bessarab, L. Gilchrist) and non-Indigenous researchers (K. Smith, D. LoGiudice, L. Flicker) who led the then development of the Good Spirit Good Life Quality-of-Life Tool and Framework published in 2019 (Smith et al., 2021).
3. **The quantitative team** comprising an Aboriginal epidemiologist (J.N. Luke) and non-Indigenous demographer (J. Temple) and non-Indigenous aging researcher (B. Dow).

In September 2022, the qualitative and quantitative teams met to select independent and dependent variables from the NATSISS to include in analyses. Variables were included based on perceived alignment between NATSISS items and factors from the Good Spirit Good Life Framework.

In March 2023, data were presented to the GSGL Elders Governance Group for their expert feedback, interpretation, and validation of findings. To support these groups’ involvement in the co-analyses, a data literacy workshop was held in Perth, Western Australia. This 2-hr workshop introduced key statistical concepts of counts, percentages, *p* values, odds ratios, and confidence intervals using easy-to-understand language, visuals, and used the preliminary study findings as examples. Elders participated in the co-analysis with the quantitative and qualitative groups by providing their interpretations and sense-making of the data and how findings related to their lived experiences as older Aboriginal and Torres Strait Islander people. There were concerns that a reliance on the published literature to interpret findings would have led to a Eurocentric interpretation of our results, so the co-analysis method was used to give voice to older people and center Aboriginal ways of knowing, doing, and being.

Members of the GSGL Elders Governance Group were financially remunerated for their time as collaborators. Recognizing that the knowledge of GSGL Elders Governance Group shaped this research, Elders were offered the opportunity to be recognized either as coauthors or in acknowledgment. Elders chose to be acknowledged.

An A2–4-page plain-language summary of research published here was provided to the GSGL Elders Governance Group as well as disseminated throughout Aboriginal and Torres Strait networks including organizations (see Author Note 2).

### Measures

Age was measured in years, with analyses restricted to older adults of ≥45 years. This age threshold is consistent with other studies that consider Aboriginal and Torres Strait Islander people’s younger age of life expectancy, younger age of onset for health conditions of aging, and younger age of eligibility to access government-supported aged care services (Gubhaju et al., 2013; Temple et al., 2019; Waugh & Mackenzie, 2011).

All survey items including Aboriginal and/or Torres Strait Islander status were self-reported by survey participants.

Independent variables relating to participation in cultural events and activities came from two questions in the NATSISS “language and culture” module. Dependent variables corresponding to areas of the Good Spirit, Good Life Framework were identified in the NATSISS and dichotomized for analysis. Independent and dependent variables and the handling of their dichotomization are presented in Table 1.

**Table 1.** Aligning the Good Spirit Good Life Framework Components With Variables From the NATSISS

Domain of Good Spirit Good Life framework	NATSISS questions	Variable label	Dichotomization of variable
Independent variables			
Culture	Types of cultural events, ceremonies, or organizations attended in last 12 months (multiple selection possible)		
	1. Ceremonies	Ceremonies	“attended”/“did not attend”
	2. Funerals/Sorry business	Funerals/Sorry business	“attended”/“did not attend”
	3. NAIDOC week activities	NAIDOC	“attended”/“did not attend”
	4. Sports carnivals (excluding NAIDOC week activities)	Sports carnival	“attended”/“did not attend”
	5. Festivals or carnivals involving arts, crafts, music, or dance (excluding NAIDOC week activities)	Festivals/carnivals	“attended”/“did not attend”
	6. Involved in Aboriginal and/or Torres Strait Islander Organizations	Involved in organizations	“attended”/“did not attend”
	7. None of the above	No events	“attended”/“did not attend”
	Types of selected cultural activities participated in the last 12 months (multiple selection possible)		
	1. Fished	Fished	“attended”/“did not attend”
	2. Hunted	Hunted	“attended”/“did not attend”
	3. Gathered wild plants/berries	Gathered wild plants/berries	“attended”/“did not attend”
	4. Made Aboriginal and/or Torres Strait Islander arts or crafts	Arts/crafts	“attended”/“did not attend”
	5. Performed any Aboriginal and/or Torres Strait Islander music, dance, or theater	Music/dance/theater	“attended”/“did not attend”
	6. Written or told any Aboriginal and/or Torres Strait Islander stories	Written/told stories	“attended”/“did not attend”
	7. None of the above	No activities	“attended”/“did not attend”
Dependent variables			
Basic needs	Whether household members ran out of money for basic Living expenses in last 2 weeks	Couldn’t pay basic	“ran out of money for basic living expenses / “did not run out of money for basic living expenses”
Family and friends	How often feels able to have a say with family and friends on important issues	Able to have a say with family and friends	“all,” “most,” “some,” “a little of the time”/“none,” “no family”
Country	Whether recognizes and area as homelands/ traditional	Recognizes area as homelands	“recognizes Country” / “doesn’t recognize Country”
Community	How often feels able to have a say within community on important issues	Able to have a say with community	“all,” “most,” “some,” “a little of the time” / “none,” “no family”
Culture	Whether identifies with clan tribal or language group	Identifies with cultural group	“identifies with clan, tribal, or language group”/ “does not identify with clan, tribal, or language group”
Health	1) Self assessed health status	Self-rated good health	“excellent,” “very good,” “good” / “fair,” “poor”
	2) Whether has been diagnosed with a long-term health	Long-term health condition	“has been diagnosed with a long-term health condition”/ “has not been diagnosed with a long-term health condition”
Supports and services	Whether has problem assessing services	Problem accessing services	“has problems accessing services” / “does not have problems accessing services,” “has not tried to access services”
Safety and security	1) Whether ever removed from natural family	Removed from family as child	“has been removed from natural family” / “has not been removed from natural family,” “refusal”
	2) Whether relatives ever removed from natural family	Family member removed from family	“relatives removed from natural family” / “relatives never removed from natural family,” “not known or not stated,” “refusal”
	3) Types of selected stressors experienced by self, family, or friends in last 12 months	Discrimination	“treated badly or discriminated” / “null response”
Spirituality	^a^		
Future planning	^a^		
Respect	^a^		
Elder role	^a^		

*Notes*: NATSISS = National Aboriginal and Torres Strait Islander Social Survey.

^a^No question/s in the NATSISS corresponding to the domains of the Good Spirit Good Life framework.

### Statistical Analyses

All analyses were conducted in Stata 16 (StatCorp, College Station, TX) via the ABS remote access Data Laboratory (DataLab, ABS, Canberra, Australia).

All categorical data have been reported as frequencies (percentages) and 95% confidence intervals and χ^2^ tests were used to assess trends in prevalence. Owing to the underlying complex survey sampling methods of the NATSISS, weighted frequencies (percentages) are presented where weighted adjustments have been used to obtain correct variance. Weighted adjustments were performed and the delete-one jack-knife method using the 250 replicate weights provided in the NATSISS data file.

Univariate logistic regression analyses were fitted using replicate jack-knife standard errors and used to calculate the odds ratios for demographic and contextual factors from the Good Spirit Good Life Framework associated with participation in each of the individual cultural events and activities.

Multivariate logistic regression was used to calculate odds ratios for participation in any event and any activity. Similarly, multivariate logistic regression models were fitted with replicate jack-knife standard errors and were adjusted for demographic and/or contextual factors.

## Results

### Characteristics of NATSISS Survey Respondents

In total, 11,178 Aboriginal and Torres Strait Islander people participated in the NATSISS.

All subsequent analyses relate to the 2,730 (24.4%) survey respondents aged ≥45 years.

Stratification of participants by sex revealed a greater proportion of female (56.0%) compared to male (44.0%) completing the survey. There was also greater representation by those aged 45–54 years (44.2%) compared to those in the older age groups of 55–64 (33.9%) and 65+ years (21.9%).

### Proportion Reporting Participation in Cultural Events and Activities

Close to two in three Aboriginal and Torres Strait Islander older adults participated in at least one cultural event and more than one in two older adults in at least one cultural activity (Table 2). Events and activities with the greatest proportion of the population partaking, included Funerals/sorry business (42.7%; see Author Note 3), NAIDOC week (see Author Note 4) (35.2%), and activity of fishing (44.6%). Events least participated included ceremonies (19.6%) and activities of performing any music/dance/theater (9.4%). See Table 1.

**Table 2.** Prevalence and Percent Participation in Cultural Events and Activities by Demographic Characteristics, 2014–2015

Variable	Age			Gender		Remoteness		Total	
	45–54	55–64	65+	Male	Female	Remote	Nonremote		
	(*n* = 1,206)	(*n* = 926)	(*n* = 598)	(*n* = 1,201)	(*n* = 1,529)	(*n* = 958)	(*n* = 1,772)		
	Weighted %	Weighted %	Weighted %	Weighted %	Weighted %	Weighted %	Weighted %	Unweighted *n* (%)	
Cultural events									
Ceremonies	22.7	17.2	15.8^*^	17.6	21.4	34.5	15.2^***^	592	(19.6)
Funerals/Sorry business	46.1	38.4^**^	40.8	38.9	45.9^**^	71.9	34.0^***^	1,286	(42.7)
NAIDOC week activities	39.8	32.0^*^	28.8^**^	29.4	40.4^***^	35.4	35.2	912	(35.2)
Sports carnivals	27.2	17.9^**^	16.3^**^	19.7	24.2	33.4	18.8^***^	586	(22.1)
Festivals/carnivals	28.3	21.4^*^	19.8^*^	19.9	28.4^**^	29.0	23.1	708	(24.5)
Involved in organizations	26.8	24.2	20.3	20.9	28.1^*^	24.1	24.9	671	(24.8)
No events	32.7	41.5^**^	45.6^***^	42.9	33.6^**^	19.3	43.5^***^	988	(38.0)
Funerals/Sorry business	46.1	38.4^**^	40.8	38.9	45.9^**^	71.9	34.0^***^	1,286	(42.7)
NAIDOC week activities	39.8	32.0^*^	28.8^**^	29.4	40.4^***^	35.4	35.2	912	(35.2)
Sports carnivals	27.2	17.9^**^	16.3^**^	19.7	24.2	33.4	18.8^***^	586	(22.1)
Festivals/carnivals	28.3	21.4^*^	19.8^*^	19.9	28.4^**^	29.0	23.1	708	(24.5)
Cultural activities									
Fished	49.0	44.6	33.3^***^	53.9	36.4^***^	58.7	40.4^***^	1,204	(44.6)
Hunted	22.7	16.6^*^	14.0^**^	24.0	14.9^***^	48.6	10.4^***^	641	(19.1)
Gathered wild plants/berries	24.2	20.4	14.2^**^	17.6	24.1^*^	34.0	17.2^***^	610	(21.1)
Arts/crafts	20.5	18.0	12.7^*^	13.7	22.1^**^	9.7	17.8	487	(18.2)
Music/dance/theater	10.9	9.3	5.8^*^	6.6	11.9^**^	11.3	8.8	246	(9.4)
Written/told stories	18.0	18.4	19.2	15.8	20.5	23.4	16.8^**^	557	(18.3)
No activities	38.2	40.0	52.2^***^	35.9	46.4^***^	25.7	46.2^***^	1,089	(41.5)

*Notes*: Age group 45–54 years is the base category for tests of proportions.

^***^ *p* < .001.

^**^ *p* < .01.

^*^ *p* < .05.

### Demographic Characteristics and Participation

Participation across all cultural events and activities was greatest among females (see Author Note 5), except for fishing and hunting where participation was higher for males. For age, there was an inverse relationship between increasing age and the proportion of older adults reporting participation, with the eldest age group of older adults less likely to participate in ceremonies, NAIDOC week, sports carnivals, festivals/carnivals, fishing, hunting, gathering wild plants/berries, arts/crafts, music/dance/theater. However, the 65+year age group had the highest proportion of individuals writing/telling stories of the three groups. The proportion of people reporting involvement in organizations did not differ across age groups.

In univariate analysis, there were regional differences with a greater proportion of respondents in remote areas partaking in ceremonies, funerals/sorry business, sports carnivals, fishing, hunting, gathering wild/plants and berries, and writing/telling stories. However, participation in NAIDOC week activities, festival/carnivals, involvement in Aboriginal or Torres Strait organizations, arts/crafts, and music/dance/theater did not differ among remote and nonremote residents.

### Good Spirit Good Life Components and Participation

Tables 3 and 4 present the proportion of older people reporting some of the components from Good Spirit Good Life Framework and present the univariate associations with participation. Most older adults were strong in identity, with most recognizing homelands (80.9%) and identifying with a cultural group (67.6%), and most reporting having a say in their community (70.0%) and with their family and friends (95.2%). Surprisingly, a majority reported good health (86.0%) despite a majority also reporting long-term health conditions (84.7%). However, negative social environments, including having been removed from natural family in childhood (14.0%), having a family member removed (41.3%), discrimination (5.3%), problems accessing services (24.6%), and being unable to pay basic living expenses (22.8%) were reported among older people.

**Table 3.** Prevalence and percent reporting components of Good Spirt Good Life Framework by Participation in Cultural Events in the Last 12 months, 2014–2015

NATSISS variable aligning with the Good Spirit Good Life Framework	Ceremonies	Funerals/sorry business	NAIDOC week	Sports carnivals	Festivals/carnivals	Involved in organizations	No events	Total	
	Weighted %	Weighted %	Weighted %	Weighted %	Weighted %	Weighted %	Weighted %	Unweighted *n* (weighted %)	
Recognizes area as homelands	23.1^***^	49.0^***^	40.2^***^	25.5^***^	28.7^***^	29.0^***^	30.5^***^	2,223	(80.9)
Identifies with cultural group	26.7^***^	54.8^***^	44.0^***^	28.2^***^	32.3^***^	32.7^***^	24.6^***^	1,884	(67.6)
Self-rated good health	20.4	43.9^**^	36.4	23.0	25.5^*^	25.2	36.8^*^	2,313	(86.0)
Long-term health condition	19.5	41.6	35.0	21.5	24.5	24.8	38.6	2,307	(84.7)
Able to have a say with family and friends	20.2^*^	43.1	35.8^*^	22.6	25.1^*^	25.3	37.3^*^	2,592	(95.2)
Able to have a say with community	24.0^***^	45.9^***^	41.8^***^	26.6^***^	30.0^***^	30.0^***^	33.3^***^	1,960	(70.0)
Removed from family as child	17.9	41.6	40.6	19.6	21.5	25.8	35.2	392	(14.0)
Family member removed from family	21.5	48.9^***^	45.3^***^	26.6^**^	31.1^***^	33.7^***^	28.5^***^	1,120	(41.3)
Couldn’t pay basic living expenses	27.2^*^	52.5^**^	34.9	26.7	25.1	29.5	30.1^*^	629	(22.8)
Problem accessing services	26.0^**^	48.2^*^	40.1^*^	29.8^**^	28.2	29.7^*^	30.0^**^	719	(24.6)
Discrimination	27.4	54.8^*^	48.2^*^	26.4	36.9^*^	46.5^***^	26.4	136	(80.9)

*Notes*: NATSISS = National Aboriginal and Torres Strait Islander Social Survey.

^***^ *p* < .001,

^**^ *p* < .01, and

^*^ *p* < .05 for participating in event to nonparticipation.

**Table 4.** Prevalence and Percent Reporting Components of Good Spirt Good Life Framework by Participation in Cultural Activities in the Last 12 months, 2014–2015

NATSISS variable aligning with the Good Spirit Good Life Framework	Fished	Hunted	Gathered wild plants/berries	Arts/crafts	Music/dance/theater	Written/told stories	No activities	Total	
	Weighted %	Weighted %	Weighted %	Weighted %	Weighted %	Weighted %	Weighted %	Unweighted *n* (weighted %)	
Recognizes area as homelands	48.2^***^	22.2^***^	24.5^***^	21.3^***^	11.3^***^	22.0^***^	36.0^***^	2,223	(80.9)
Identifies with cultural group	50.0^***^	24.2^***^	28.4^***^	24.7^***^	13.1^***^	25.6^***^	32.4^***^	1,884	(67.6)
Self-rated good health	46.9^***^	20.0	21.4	18.9	9.7	18.9	39.3^***^	2,313	(86.0)
Long-term health condition	44.1	17.5^**^	21.3	19.2^*^	9.9	18.8	41.6	2,307	(84.7)
Able to have a say with family and friends	45.3^*^	19.5	21.5	18.5	9.5	18.6	40.9	2,592	(95.2)
Able to have a say with community	48.0^**^	21.6^**^	25.3^***^	22.4^***^	11.9^***^	22.3^***^	36.5^***^	1,960	(70.0)
Removed from family as child	44.1	17.5^**^	21.3	19.2^*^	9.9	18.8	41.6	392	(14.0)
Family member removed from family	42.5	22.6^**^	24.7^*^	23.6^**^	11.3	26.1^***^	38.8	1,120	(41.3)
Couldn’t pay basic living expenses	45.5	24.4^**^	26.2	21.3	14.8^*^	21.7	35.9	629	(22.8)
Problem accessing services	49.6	26.8^***^	26.9^**^	23.6^**^	10.4	27.1^***^	32.4^***^	719	(24.6)
Discrimination	42.5	22.6^**^	24.7^*^	23.6^**^	11.3	26.1^***^	28.8	136	(5.3)

*Notes*: NATSISS = National Aboriginal and Torres Strait Islander Social Survey.

^***^ *p* < .001,

^**^ *p* < .01, and

^*^ *p* < .05 for participating in activity to nonparticipation.

In univariate analyses, older people recognizing homelands, identifying with a cultural group, or reporting having a say in community, had significantly higher participation across most cultural events and activities, as did those reporting discrimination or family member removed. The reporting of good health and absence of a long-term condition was generally associated with greater participation in cultural events and activities, with the exception of arts/crafts and writing/telling stories, where the opposite was found where having a long-term condition was associated with a higher frequency of participation with this significant for arts/crafts only. For older adults removed from natural family in childhood, the proportion participating in NAIDOC activities, involvement in organizations, and writing stories was higher than those not removed; however, these were not statistically meaningful. For older people, not being able to pay basic living expenses or access services is associated with greater participation in almost all the events and activities, with many of these associations significant.

### Demographic and Good Spirit Good Life Components Associating With Each Event and Activity

Univariate analyses presented in Supplementary Table 1 reveal that the demographic and Good Spirit Good Life components reported by survey respondents varied across each of the six activities and six events. Males had twofold greater odds of participating in fishing and hunting, while significant lower odds of attending sorry business, NAIDOC, festivals/carnivals, gathering wild plants/berries, arts/crafts, and music/dance/theater. Both 55–64- and 65+-year-old groups reported lower participation in Sports carnivals than the 45–54-year-old group, with the 65+ group also reporting lower participation in fishing and gathering wild/plants or berries relative to the youngest group. The 65+ group did, however, have 56% greater odds of writing/telling stories than the youngest group. Those in remote areas had greater odds for participation in ceremonies funerals/sorry business, sports carnival, fishing, hunting, gathering wild plants/berries; however, participation in NAIDOC, organizations, festivals or carnivals and activities of arts/crafts, music/dance/theater, and written/told stories did not differ by remoteness. Older people recognizing homelands and those identifying with cultural groups had higher odds or participation across all events and activities. Fishing was the only event or activity associated with self-reported good health. Being able to have a say in community was associated with higher odds of participation in most events or activities, as was having a family member removed. Participating in organizations and gathering wild berries/plants associated with higher odds of also reporting discrimination.

### Multivariate Models: Participation in Any Cultural Event and Any Activity

Multivariate analyses for the participation in any event or any activity are presented in Table 5, with findings relatively consistent across the models. In all models, males had lower odds for participation in any event, but higher odds for participating in any activities, whereas older people in remote areas consistently had 2–3 odds greater participation in events and activities compared to older people living nonremote. The older 65+ age group had lower odds of participating in events and activities in all models except in Model 4, where accounting for the contribution of all demographic and Good Spirit Good Life components, this association between increasing age and lower participation in any events was not significant. Good Spirit Good Life components of recognizing area as homelands, identifying with cultural groups, and having a say in community were associated with participation in any event or any activity in all models, while having a family member removed was associated with the odds of participating in an event only. Conversely, not being able to pay basic living expenses is associated with greater odds of participating in an activity.

**Table 5.** Associations Between Participation in Any Cultural Events or Activities and Demographic and Components of the Good Spirit Good Life Framework, Multivariate Analysis Using Logistic Regression

Variable	Model 1	Model 2	Model 3	Model 4
	Demographic	GSGL variables (individual and community)	Demographic and GSGL variables (individual and community)	Demographic and GSGL variables (individual, community, and societal)
	OR* (95% CI)	OR* (95% CI)	OR* (95% CI)	OR* (95% CI)
Participated in events in the last 12 months				
Age				
45–54	1	—	—	1
55–64	0.67 (0.50, 0.90)	—	0.84 (0.61, 1.16)	0.88 (0.62,1.24)
65+	0.55 (0.40, 0.77)	—	0.63 (0.44, 0.91)	0.69 (0.48, 1.01)
Sex (male)	0.65 (0.50, 0.86)	—	0.63 (0.47, 0.84)	0.65 (0.48, 0.88)
Remote	3.32 (2.35, 4.70)	—	2.68 (1.88, 3.81)	2.71 (1.88, 3.90)
Recognizes area as homelands	—	4.15 (3.00, 5.73)	2.46 (1.67, 3.61)	2.39 (1.60, 3.55)
Identifies with cultural group	—	2.63 (1.80, 3.83)	3.90 (2.78, 5.46)	3.56 (2.47, 5.14)
Self-rated good health	—	1.06 (0.70, 1.61)	1.03 (0.67, 1.58)	1.13 (0.73, 1.76)
Long-term health condition	—	0.83 (0.56, 1.23)	0.89 (0.60, 1.34)	0.85 (0.55, 1.30)
Able to have a say with family and friends	—	1.20 (0.65, 2.22)	1.29 (0.67, 2.51)	1.27 (0.62, 2.61)
Able to have a say with community	—	1.52 (1.11, 2.09)	1.55 (1.11, 2.15)	1.57 (1.11, 2.23)
Removed from family as child	—	—	—	0.96 (0.64, 1.44)
Family member removed from family	—	—	—	1.67 (1.17, 2.40)
Couldn’t pay basic living expenses	—	—	—	1.35 (0.97, 1.88)
Problem accessing services	—	—	—	1.17 (0.76, 1.80)
Discrimination	—	—	—	0.89 (0.48, 1.62)
Participated in activities in the last 12 months				
Age				
45–54	1	—	1	1
55–64	0.92 (0.67, 1.25)	—	1.10 (0.79, 1.54)	1.12 (0.80, 1.58)
65+	0.55 (**0.39, 0.77**)	—	0.61 (**0.43, 0.85**)	0.64 (**0.45, 0.91**)
Sex (male)	1.56 (**1.19, 2.04**)	—	1.69 (**1.27, 2.24**)	1.72 (**1.29, 2.30**)
Remote	3.32 (**2.35, 4.70**)	—	2.68 (**1.88, 3.81**)	2.71 (**1.88, 3.90**)
Recognizes area as homelands	—	4.15 (**3.00, 5.73**)	2.46 (**1.67, 3.61**)	2.39 (**1.60, 3.55**)
Identifies with cultural group	—	2.63 (**1.80, 3.83**)	3.90 (**2.78, 5.46**)	3.56 (**2.47, 5.14**)
Self-rated good health	—	1.06 (0.70, 1.61)	1.03 (0.67, 1.58)	1.13 (0.73, 1.76)
Long-term health condition	—	0.83 (0.56, 1.23)	0.89 (0.60, 1.34)	0.85 (0.55, 1.30)
Able to have a say with family and friends	—	1.20 (0.65, 2.22)	1.29 (0.67, 2.51)	1.27 (0.62, 2.61)
Able to have a say with community	—	1.52 (**1.11, 2.09**)	1.55 (**1.11, 2.15**)	1.57 (**1.11, 2.23**)
Removed from family as child	—	—	—	0.96 (0.64, 1.44)
Family member removed from family	—	—	—	1.67 (**1.17, 2.40**)
Couldn’t pay basic living expenses	—	—	—	1.35 (0.97, 1.88)
Problem accessing services	—	—	—	1.17 (0.76, 1.80)
Discrimination	—	—	—	0.89 (0.48, 1.62)

*Notes*: CI = confidence interval; GSGL = Good Spirit Good Life; OR = odds ratio.

^a^Weighted odds ratios. Bold denotes confidence intervals are significant.

## Discussion

Grounded in Indigenous theories for “ageing well” and drawing on culturally validated frameworks such as the Good Spirit Good Life Framework, our research measures social participation that is relevant for older Aboriginal and Torres Strait Islander people (Smith et al., 2021).

For participation, we found that a sizable majority of older Aboriginal and Torres Strait Islander people participated in cultural events (62.0%) and activities (58.5%). Older people were also strong in their cultural identities that connected them not only to their homelands and Country, but to their clan, tribal, or language groups. The GSGL Elders Governance Group members provided validation to study measures as they spoke about their own participation in the measured cultural events and activities. The Elders spoke of their cultural responsibilities as board members of organizations and of their attendance at NAIDOC events, sporting carnivals, festivals, and sadly too many funerals. GSGL Elders Governance Group members spoke of family members who fished, hunted, and collected plants/berries, and how this connected them to Country, families, and communities, but emphasized that fishing, hunting, and collecting plants/berries also had a larger purpose of providing food or medicine. As we consider participation in events and activities, these are collectivist pursuits in that they connect Aboriginal and Torres Strait Islander people and bring benefits to more than the individual (Gibson et al., 2020; Smith et al., 2021).

We found gendered differences in participation, where the female participants had higher odds for participating in most events and activities, with the exception of fishing and hunting. The GSGL Elders Governance Group who were all female, laughed at this finding telling us to look around the room. Although Elders noted that men often sat in groups and organizations, men often needed safe space such as community centers and men’s only spaces to feel comfortable to engage. In talking to fishing and hunting, these were framed as traditionally masculine activities, where men in the family would fish together, but the whole family would eat together. Elders commented that more should be done with aged care to better integrate fishing and hunting, and the inclusion of such foods.

Cultural events and activities were attended with greater frequency in remote areas. Not surprising, as these were the last known areas to see the colonial frontier, where disruptions to sociocultural livelihood were less pronounced. The GSGL Elders Governance Group members, who lived in urban areas and spoke about colonization and the lost opportunities to fish, hunt, and collect plants/berries as Country had changed. They told us how rivers and lands were no longer viable food sources and how it was easier to get other options such as takeaway and Uber Eats. Elders reflected that many traditional ways of participating in culture such as ceremonies had been disrupted for those who grew up in cities and who were forbidden to practice their cultures. However, we found that people in nonremote and remote areas participated with similar frequency are activities of NAIDOC week, festivals/carnivals, involvement in organizations, arts/crafts, music/dance/theatre, and writings/telling of stories. These events and activities are all modern articulations of culture, where traditional cultural knowledge is not a requirement for participation. Many of the Aboriginal and Torres Strait Islander organizations were founded in the 1970s in urban and regional areas as sites of connection so it is not surprising that older people from urban areas were involved in organizations (Waugh & Mackenzie, 2011).

With consideration to age, the 65+-year-old age group was less likely to participate in most events and activities with the exception of funerals/sorry business, involvement in organizations, and writing/telling stories. The GSGL Elders Governance Group in interpreting this finding spoke of the unique cultural role of Elders (who tend to be over 65 years), of their responsibilities to communities, and how their lived experience and cultural knowledge are valued in organizations. Elders also spoke of the role of Elders in passing down knowledge and how writing stories was a way of passing down their stories and experiences to younger generations. This role in the passing of stories, particularly of the activism and advocacy of family and community, is reported as allowing younger people to grow their cultural knowledge and awareness of their story and of wider Aboriginal history (Gibson et al., 2020). Participation in both sporting events and carnivals/festivals was also framed in terms of connecting across generations, with attendance as spectators seen as a way of supporting grandchildren and great-grandchildren (Gair et al., 2019; Smith et al., 2021). It is through their roles as educators, advisors, and grandparents of younger people that older Aboriginal and Torres Strait Islander people acquire social capital within their communities (Warburton & McLaughlin, 2006).

In terms of social characteristics, our data revealed that older people have been affected by ongoing colonialism. Older Aboriginal and Torres Strait Islander people reported experiences of removal from their natural family in childhood, having family members removed, experiencing discrimination, as well as issues around poverty and access to services. Elders on the GSGL Elders Governance Group spoke often about a lack of monetary resources and how, in some instances, this was a barrier to participation, but in other instances resulted in greater participation. For example, the GSGL Elders Governance Group spoke of a lack of transport and funds as a barrier to attending funerals. Conversely, lower incomes and not being able to meet basic living expenses meant that participating in fishing, hunting, and gathering were ways of obtaining food in times of financial crisis. Elders also spoke of their responsibilities and obligations in supporting younger members financially.

A majority of older people reported good health (86.0%), despite many also reporting a long-term health condition (84.7%). This paradox is best understood in the definitions of Aboriginal and Torres Strait Islander health where “health means not just the physical well-being of an individual but refers to the social, emotional and cultural well-being of the whole community in which each individual is able to achieve their full potential as a human being thereby bringing about the total well-being of their community contextualised through definitions of health” (National Aboriginal Community Controlled Health Organisation, 2013). Good health was associated with greater odds of participation in events and activities (with this significant for activities only). However, owing to the cross-sectional nature of the survey, we are unable to say if good health leads to participation or if being involved in events and activities promotes health. Although, the benefits associated with participation and having a good spirit are recognized in the literature, so too is the impact that poor health and well-being can have on fulfilling cultural roles and participation (Coombes et al., 2018; Gibson et al., 2020; Smith et al., 2021; Waugh & Mackenzie, 2011).

What these data do show is that there are many types of cultural events and activities that older Aboriginal and Torres Strait Islander people participate in. Our data demonstrating variation in the types of events and activities participated in by demographic and social factors also challenges the notion of a homogenous Aboriginal and Torres Strait Islander culture and is a revelation of the diversity among this population. Our findings of demographic variations in participation by age, sex, and location suggest that any approach to support Aboriginal and Torres Strait Islander older people’s participation in culture must consider the demographic of each specific population.

### Strengths

Lovett et al. (2020) contest that “who determines what knowledge is - and who has the right to speak to that knowledge - is important” (Lovett et al., 2020). A key strength of our paper is that Aboriginal voices, including those of older Aboriginal and Torres Strait Islander Elders, have been instrumental in shaping the knowledge about older Aboriginal and Torres Strait Islander people generated in this paper.

Our participatory methodology has enabled the meaningful and significant contribution of older people to this research in a couple of ways. First, our framework for analysis drew on the 12 factors of the culturally validated Good Spirit Good Life Quality-of-life tool, a tool that was developed by Smith et al. using participatory action research where lived experience, knowledge, and wisdom of Elders and older people is captured (Smith et al., 2021). Second, through our triangulation approach, Aboriginal and non-Indigenous researchers were able to work with the GSGL Elders Governance Group Elders to get first-hand interpretations and meaning-making from the data that reflected older people’s understandings of their sociocultural worlds informed by their lived experience and realities. These expert voices contributed to self-representation in the data, allowing Elders to control the data narrative around what is presented about them. These aspects of our methodology mean that potential cultural biases arising from “outsider” construction are largely mitigated with confidence in the internal validity of our findings (Lock et al., 2021).

Quayle et al. (2019) emphasize the importance of research narratives promoting cultural resistance and survival as these provide counter-discourses to long-perpetuated deficit narratives of older Aboriginal and Torres Strait Islander peoples that describe them as dysfunctional, diseased, ill, and wounded (Quayle & Sonn, 2019). While research frames must also acknowledge “pluralities of perspective” where processes of Aboriginal resilience, strength, and well-being also intersect with complex processes of colonialism (Hatala et al., 2016). Recognizing this, our analysis deliberately used a strengths-based framework where Aboriginal and Torres Strait Islander culture and community were seen as strengths that enable older people to “ageing well” while also recognizing there are complex historical and contemporary social realities that also shape participation in culture and communities.

A limitation of many nationwide social survey instruments is that they assume a universality of social experiences, often privileging sociocultural characteristics of dominant groups within a country. A strength of the NATSISS is that it was developed in consultation with Aboriginal peoples with survey items deliberately identified that would measure social dimensions that are unique and important to the lives of Aboriginal and Torres Strait Islander people. This means that we were able to measure and report rich contextual information, including aspects of identity, place, social interactions, and participation that are often omitted from social research (Walter & Andersen, 2013). Another strength of our analysis is that the NASTSISS has allowed us to draw on a nationally representative and powered sample which means that the findings are likely to be generalizable beyond the survey context.

### Limitations

As we highlight the strengths of our methodology, it is also important to recognize some key limitations. First, there are limitations to applying quantitative methods to understand sociocultural dimensions relating to Aboriginal and Torres Strait Islander people’s lives. Second, there are also limitations with the NATSISS as a survey instrument.

A key limitation of the NATSISS is that it was not specifically developed as a survey for older people. As a household survey for people ≥15 years of age residing in private dwellings only, the NATSISS does not include people who are homeless, in hospitals, or nursing homes, meaning that there are key older groups missing from our analysis. Omission of these individuals means that rates of long-term conditions and those able to meet basic needs are likely underestimated and participation in events and activities overestimated. There is also the issue of the cross-sectional design of the NATSISS, which means we are unable to investigate directions of causality.

We also note that survey items within the NATSISS are somewhat derivative, in that complex and nuanced sociocultural phenomena related to country, identity, and community are reduced into measurable abstract units (Walter & Andersen, 2013). The NATSISS also codifies people of diverse histories and cultures from over 250+ nations into a category of “Aboriginal and Torres Strait Islander” who participate in “Aboriginal and Torres Strait Islander cultural” activities and events. We are mindful that the application of this imposed social term and broad characterization of culture can be problematic and essentializing.

As we consider the Good Spirit Good Life framework, there were no NATSISS items that captured factors of respect, Elder role, spirituality, and future planning. This means there are sociocultural phenomena relating to older people’s lives that are important to them that we have been unable to explore. These factors could be considered in future NATSISS surveys or conversely, a survey specific to older people may provide more complete data providing detailed insights.

We are also mindful that the NATSISS was conducted in 2014–2015 pre-COVID-19 pandemic, and current rates of participation should be measured in subsequent iterations of the NATSISS.

## Conclusion

Aboriginal and Torres Strait Islander older people widely participate in their communities. This research highlights the importance and assists in validating the significance of the Good Spirit Good Life tool as a culturally relevant framework for measuring the quality of life for older Aboriginal and Torres Strait Islander peoples across Australia. Equally, they highlight the importance of social surveys measuring the sociocultural factors that are specific to Aboriginal and Torres Strait Islander people’s lives. These data can help to inform organizations who are providing services and support families who are caring for older Aboriginal and Torres Strait Islander members on what is required to ensure that older members of our families and communities are supported to live a good life.

## Supplementary Material

gbae100_suppl_Supplementary_Figure_S1

gbae100_suppl_Supplementary_Table_S1

## Author Notes

1.Terminology contradictory to the “Reframing Aging Journal Manuscript Guidelines”: The cultural term “Elders” is used throughout this manuscript. In Aboriginal and Torres Strait Islander contexts, “Elders” is a cultural term used with respect to describe older adults who are cultural leaders in their communities and who are recognized for their wisdom, knowledge, and experience.

2.Aboriginal and/or Torres Strait Islander Organizations refer to the community-controlled health, social, and cultural organizations set up by and for Aboriginal people.

3.Sorry business is a cultural term used to describe the mourning period and the process and practices that take place after the death of a family or community member.

4.NAIDOC stands for National Aborigines and Islanders Day Observance Committee, and NAIDOC week refers to the week of celebrations each July where Aboriginal and Torres Strait Islander people come together to celebrate history, culture, and achievements.

5.Terms “male” and “female” are used throughout this study as the survey asked respondents to tick sex as either “male” or “female.”
