# Supplementary material for: Counting the Ways That Aboriginal and Torres Strait Islander Older People Participate in Their Communities and Culture
Source: J Gerontol B Psychol Sci Soc Sci. 2024 May 31;79(8):gbae100. doi: 10.1093/geronb/gbae100 (PMC11234290; doi:10.1093/geronb/gbae100)
Supplement: gbae100_suppl_Supplementary_Figure_S1 [file gbae100_suppl_supplementary_figure_s1.docx]

Supplementary Figure 1: the Good Spirit Good Life Framework from Smith et al (2020)
