# Supplementary material for: Counting the Ways That Aboriginal and Torres Strait Islander Older People Participate in Their Communities and Culture
Source: J Gerontol B Psychol Sci Soc Sci. 2024 May 31;79(8):gbae100. doi: 10.1093/geronb/gbae100 (PMC11234290; doi:10.1093/geronb/gbae100)
Supplement: gbae100_suppl_Supplementary_Table_S1 [file gbae100_suppl_supplementary_table_s1.docx]

**Supplementary Table 1: Associations between participation in each cultural event or activity and demographic and Good Spirit Good Life components, Univariate analysis using logistic regression.**

Participated in cultural events in the last 12 months

Ceremonies Funerals/ NAIDOC week Sports Festivals/ Involved in No events

Sorry business activities carnivals carnivals organisation

OR* (95%CI) OR* (95%CI) OR* (95%CI) OR* (95%CI) OR* (95%CI) OR* (95%CI) OR* (95%CI)

Age 45-54 1 1 1 1 1 1 1

55-64 0.82 (0.53, 1.25) 0.90 (0.65, 1.24) 0.82 (0.58, 1.16) 0.65 (**0.46, 0.93**) 0.82 (0.56, 1.20) 1.12 (0.75, 1.68) 1.14 (0.81, 1.61)

65+ 0.80 (0.51, 1.25) 1.04 (0.70, 1.56) 0.70 (0.48, 1.02) 0.62 (**0.41, 0.92**) 0.73 (0.50, 1.08) 0.95 (0.62, 1.46) 1.44 (0.99, 2.10)

Sex (male) 0.79 (0.56, 1.13) 0.73 (**0.56, 0.96**) 0.61 (**0.45, 0.84**) 0.80 (0.57, 1.13) 0.64 (**0.45, 0.90**) 0.75 (0.53, 1.08) 1.54 (**1.13, 2.09**)

Remote 2.35 (**1.60, 3.44**) 4.62 (**3.31, 6.45**) 0.81 (0.54, 1.21) 1.82 (**1.21, 2.74**) 1.12 (0.76, 1.65) 0.76 (0.53, 1.07) 0.37 (**0.26, 0.53**)

Recognises area as homelands 2.58 (**1.04, 6.40**) 2.26 (**1.37, 3.71**) 2.36 (**1.50, 3.72**) 2.01 (**1.05, 3.85**) 2.68 (**1.37, 5.26**) 2.62 (**1.29, 5.32**) 0.42 (**0.28, 0.62**)

Identifies with cultural group 4.43 (**2.33, 8.44**) 3.66 (**2.37, 5.65**) 2.54 (**1.76, 3.67**) 2.26 (**1.37, 3.71**) 3.44 (**2.20, 5.38**) 3.48 (**2.13, 5.66**) 0.28 (**0.19, 0.40**)

Self-rated good health 1.22 (0.67, 2.23) 1.25 (0.87, 1.79) 1.14 (0.71, 1.84) 1.07 (0.64, 1.79) 1.11 (0.70, 1.75) 1.00 (0.60, 1.69) 0.88 (0.57, 1.37)

Long-term health condition 1.00 (0.67, 1.50) 0.78 (0.52, 1.15) 0.89 (0.60, 1.31) 0.82 (0.52, 1.29) 1.01 (0.68, 1.51) 0.93 (0.60, 1.46) 1.18 (0.77, 1.80)

Able to have a say with family & friends 1.91 (0.63, 5.73) 1.30 (0.67, 2.54) 0.89 (0.44, 1.81) 1.23 (0.53, 2.82) 1.22 (0.48, 3.14) 1.14 (0.51, 2.56) 0.79 (0.38, 1.62)

Able to have a say with community 2.54 (**1.47, 4.38**) 1.23 (0.88, 1.71) 2.61 (**1.76, 3.87**) 2.47 (**1.56, 3.90**) 2.74 (**1.82, 4.13**) 2.81 (**1.75, 4.51**) 0.64 (**0.45, 0.90**)

Removed from family as child 0.89 (0.56, 1.40) 0.83 (0.55, 1.24) 1.15 (0.78, 1.69) 0.76 (0.50, 1.17) 0.70 (0.45, 1.07) 0.83 (0.56, 1.23) 1.04 (0.70, 1.55)

Family member removed from family 1.01 (0.70, 1.44) 1.45 (**1.08, 1.95**) 1.61 (**1.13, 2.29**) 1.36 (0.96, 1.94) 1.48 (**1.02, 2.15**) 1.77 (**1.25, 2.52**) 0.60 (**0.42, 0.86**)

Couldn’t pay basic living expenses 1.41 (0.98, 2.03) 1.00 (0.74 ,1.36) 1.32 (0.96, 1.81) 1.61 (**1.10, 2.36**) 1.22 (0.85, 1.75) 1.29 (0.92, 1.80) 0.74 (0.53, 1.03)

Problem accessing services 1.69 (**1.02, 2.79**) 1.45 (0.92, 2.29) 0.82 (0.54, 1.26) 1.18 (0.64, 2.16) 0.90 (0.53, 1.52) 1.25 (0.74, 2.11) 0.86 (0.56, 1.32)

Discrimination 1.25 (0.68, 2.28) 1.12 (0.65, 1.93 1.23 (0.72, 2.09) 0.94 (0.51, 1.71) 1.36 (0.83, 2.21) 1.95 (**1.08, 3.52**) 1.13 (0.62, 2.07)

Participated in cultural activities in the last 12 months

Fished Hunted Gathered wild Arts/ Music/dance Written/ No activities

Plants/ berries Crafts /theatre told stories

OR* (95%CI) OR* (95%CI) OR* (95%CI) OR* (95%CI) OR* (95%CI) OR* (95%CI) OR* (95%CI)

Age 45-54 1 1 1 1 1 1 1

55-64 0.90 (0.66, 1.23) 0.71 (0.49, 1.04) 0.95 (0.61, 1.46) 1.06 (0.71, 1.56) 1.01 (0.55, 1.86) 1.32 (0.93, 1.89) 0.89 (0.63, 1.25)

65+ 0.53 (**0.37, 0.77**) 0.66 (0.42, 1.04) 0.59 (**0.39, 0.92**) 0.66 (0.41, 1.05) 0.61 (0.31, 1.18) 1.56 (**1.02, 2.39**) 1.56 (**1.10, 2.21**)

Sex (male) 2.14 (**1.63, 2.82**) 2.39 (**1.71, 3.35**) 0.70 (**0.49, 0.99**) 0.59 (**0.41, 0.85**) 0.55 (**0.33, 0.93**) 0.84 (0.58, 1.21) 0.58 (**0.44, 0.77**)

Remote 1.82 (**1.25, 2.65**) 8.41 (**5.64, 12.54**) 2.15 (**1.47, 3.12**) 0.91 (0.61, 1.36) 1.05 (0.59, 1.85) 1.17 (0.83, 1.66) 0.49 (**0.35, 0.67**)

Recognises area as homelands 1.61 (**1.05, 2.48)** 2.00 (**1.05, 3.80**) 1.83 (**1.03, 3.28**) 2.17 (**1.15, 4.09**) 4.23 (**1.61, 11.13**) 3.75 (**1.86, 7.58**) 0.53 **(0.36, 0.79**)

Identifies with cultural group 1.52 (**1.04, 2.23**) 1.71 (**1.16, 2.52**) 4.10 (**2.62, 6.40**) 4.41 (**2.75, 7.07**) 5.46 (**2.25, 13.29**) 5.81 (**2.99, 11.28**) 0.46 (**0.32, 0.66**)

Self-rated good health 1.73 (**1.13, 2.63**) 1.28 (0.71, 2.30) 0.87 (0.51, 1.47) 1.25 (0.74, 2.12) 1.11 (0.50, 2.46) 1.32 (0.78, 2.20) 0.60 (**0.41, 0.88**)

Long-term health condition 1.08 (0.69, 1.69) 0.61 (0.36, 1.03) 1.13 (0.75, 1.71) 1.58 (0.98, 2.54) 1.37 (0.74, 2.54) 1.13 (0.70, 1.81) 0.87 (0.56, 1.36)

Able to have a say with family & friends 1.32 (0.64, 2.70) 1.72 (0.54, 5.50) 1.22 (0.53, 2.80) 0.70 (0.30, 1.60) 0.63 (0.20, 2.01) 0.81 (0.28, 2.33) 1.02 (0.52, 1.99)

Able to have a say with community 1.32 (0.94, 1.85) 1.67 (**1.12, 2.49**) 2.42 (**1.63, 3.60**) 2.75 (**1.70, 4.46**) 3.64 (**1.89, 7.01**) 2.65 (**1.74, 4.03**) 0.58 (**0.42, 0.80**)

Removed from family as child 0.83 (0.56, 1.21) 0.77 (0.47, 1.26) 0.77 (0.48, 1.24) 0.98 (0.63, 1.53) 1.55 (0.85, 2.82) 1.09 (0.71, 1.68) 0.95 (0.62, 1.46)

Family member removed from family 0.88 (0.65, 1.19) 1.91 (**1.38, 2.66**) 1.14 (0.79, 1.65) 1.32 (0.86, 2.02) 0.93 (0.49, 1.74) 1.81 (**1.27, 2.60**) 0.93 (0.69, 1.25)

Couldn’t pay basic living expenses 1.24 (0.89, 1.72) 1.44 (0.94, 2.21) 1.26 (0.86, 1.84) 1.38 (0.94, 2.03) 0.93 (0.52, 1.67) 1.90 (**1.25, 2.88**) 0.65 (**0.46, 0.90**)

Problem accessing services 0.94 (0.63, 1.41) 1.40 (0.96, 2.05) 1.19 (0.71, 2.00) 1.02 (0.62, 1.70) 1.81 (0.87, 3.77) 1.22 (0.81, 1.82) 0.89 (0.60, 1.30)

Discrimination 0.92 (0.55, 1.54) 1.78 (0.84, 3.79) 2.23 (**1.25, 3.98**) 1.75 (0.93, 3.29) 1.65 (0.75, 3.62) 1.63 (0.86, 3.09) 0.99 (0.56, 1.76)

*Weighted odds ratios, Bold denotes confidence intervals are significant
